# Assessing the Anthropometric Profile of Spanish Elite Reserve Soccer Players by Playing Position over a Decade

**DOI:** 10.3390/ijerph17155446

**Published:** 2020-07-28

**Authors:** Jon Manuel Vega, Asier Gonzalez-Artetxe, Jon Ander Aguinaco, Asier Los Arcos

**Affiliations:** 1 Department of Physical Education and Sport, University of the Basque Country UPV/EHU, 01007 Vitoria-Gasteiz, Spain; jonmanuel10@gmail.com (J.M.V.); j.aguinaco96@gmail.com (J.A.A.); 2 Society, Sports and Physical Exercise Research Group (GIKAFIT), Department of Physical Education and Sport, University of the Basque Country UPV/EHU, 01007 Vitoria-Gasteiz, Spain

**Keywords:** team sport, talent, body composition, weight, body fat

## Abstract

The aims of this study were to describe the evolution of the anthropometric profile of soccer players over a decade and to compare the anthropometric profiles of players promoted from an elite reserve team to high-level soccer with those players who were not promoted. We examined the body mass, height, body-mass index, and body fat of 98 players enrolled in the reserve team from 2008 to 2018. The players were classified in terms of (a) the highest competitive level they achieved up to the 2019/2020 season (i.e., Spanish 1st–2nd divisions or semi-professional); (b) the period in which they played their last season on the team; and (c) their playing position. Over time, the height of goalkeepers, lateral midfielders, and attackers has increased (effect size = 0.66 ± 1.13) but has decreased in central midfielders (effect size = 0.83). The body fat of defenders has also fallen (effect size = 0.55 ± 0.95). Spanish high-level goalkeepers, lateral midfielders, and attackers were taller than their semi-professional player counterparts (effect size = 1.20 ± 1.98). Body fat did not determine promotion from a reserve team to high-level soccer, but height may be an advantage for several playing positions. The assessment of the anthropometric profile and the application of interventions should be designed according to the playing position.

## 1. Introduction

Because the financial resources of elite soccer clubs are not evenly distributed and the transfer of qualified players is a very high economic expense, less wealthy clubs must adopt different strategies in order to survive in this competitive industry [1]. One such strategy is to invest in soccer academies [2]. Such academies identify potential youth soccer players and incorporate them into their club’s youth teams with the aim of developing them into top-class soccer players [3].

Evaluating the anthropometric profile of players is common practice in soccer academies in order to optimize physical fitness performance and reduce injuries. Body fat levels correlate negatively with aerobic fitness [4] and with the sprint decrement score (observed during a repeated-sprint ability test in soccer players [5]). Similarly, an increased body-mass index and a low fat percentage have been identified as anthropometric injury risk factors in elite-standard young soccer players (under-11s to under-19s) [6]. In addition, anthropometric characteristics could determine the selection of players and their progression to the professional level [7]. Many studies have assessed the relevance of anthropometric profiles in the selection process of young soccer players [8,9]. It appears that the most talented young players (from the under-9s to under-19s) tend to be heavier and taller, as well as having more advanced skeletal maturation [8]. Elite soccer players have also been found to have a lower percentage of body fat compared with players at the lower competition levels [10]. However, despite the transition from reserve team to high-level soccer being seen as a pivotal [2] and critical [11] stage, little is known about the relevance of anthropometric factors in the final step to high-level soccer [12]. To our knowledge, only one other study has compared the anthropometric profile of players promoted from an elite reserve team to Spanish elite professional soccer (LFP players) with those not promoted (non-LFP players) [12]. The authors of that paper found differences in the height and weight values between the LFP and non-LFP players in several playing positions [12]. However, the study was carried out exclusively in a high-level club. Moreover, relevant anthropometric characteristics in soccer, such as body fat and the body-mass index [4,5,6], were not considered. Thus, further research into body fat and the body-mass index is necessary to assess the impact of anthropometric profiles on promotion from an elite reserve team to the professional level.

The physical demands in elite soccer matches have increased in recent years [13], while the physical fitness performance of the professional and reserve team soccer players has varied little over different periods [12,14,15]. Similarly, several studies have found that the anthropometric profile of youth soccer players in elite soccer academies has also barely changed [16,17]. For example, one investigation found no significant differences in the anthropometric profile (i.e., body mass, height, and body-mass index) of senior and junior Norwegian soccer players over time [14]. However, to our knowledge no study has examined the evolution of the anthropometric profile in elite reserve soccer players over a long period of time. Doing so may allow for a link to be drawn between the evolution of the anthropometric profile and the characteristics of those players who are promoted to high-level soccer.

Several studies have compared the anthropometric profile of senior soccer players according to their competition level [18,19,20]. However, none of these compared profiles in terms of the playing position of the players [18,19,20]. Given that the anthropometric profile varies substantially between playing positions [10,19,21,22], and given that the physical, physiological, and perceived demands in matches [23,24,25] (and changes over recent years [23]) have also been found to differ substantially according to playing position, comparison of anthropometric profiles in accordance with this factor should be carried out.

Therefore, the aims of this study were to evaluate whether there were any changes in the players’ anthropometric profile over a decade (i.e., 2008/2009–2017/2018), and to compare the anthropometric profile of Spanish elite reserve soccer players according to the competitive level they subsequently achieved.

## 2. Materials and Methods

### 2.1. Participants

We took anthropometric measurements for a reserve soccer team of a first-division Spanish club between the 2008/2009 and 2017/2018 seasons. Ninety-eight (age = 21.3 years ± 1.8) young professional players took part in the study, being evaluated at least twice during their last season with the team. The averages of all the assessments for each anthropometric measure (i.e., body mass, height, body-mass index, and body fat) were computed for each player (3 ± 1 pieces of data per player). After studying their sporting careers up to the beginning of the 2019/2020 season, we classified the players according to the highest competitive level they had achieved [12,15]: (i) Spanish LFP (LFP), comprising players who had signed a contract with a Spanish first- or second-division team and who had played at least one match as a full-time professional; or (ii) semi-professional (non-LFP), comprising players who had never competed in the Spanish first or second division. Thirty-nine of the forty-one players promoted to the 1st team of the club (i.e., 95%) came directly from the reserve team. The 10-year period examined was divided into two five-year terms: Period 1 (Period-1): 2008/2009–2012/2013; and Period 2 (Period-2): 2013/2014–2017/2018. Each player was assigned to the period in which he had played his last season in the reserve team. In addition, players were classified according to their playing position [12,15,26]: (a) goalkeepers; (b) defenders—lateral defenders and central defenders; (c) midfielders—lateral midfielders and central midfielders); and (d) attackers. The allocation of the playing positions was carried out by the team’s technical staff. To decide on the few controversial cases, the playing position in which the player competed more times was considered. This study was performed in accordance with the Declaration of Helsinki and approved by the Ethics Committee of the University of the Basque Country (UPV/EHU), reference number M10_2018_181.

### 2.2. Measures

According to the protocols established by the club over the last 10 years, anthropometric measurements (i.e., body mass, height, and body fat) were taken four times during each season: (i) one month before the competition period (i.e., July/August); (ii) one month after the start of the competition period (i.e., October/November); (iii) in the middle of the season (i.e., January/February); and (iv) at the end of the season (i.e., April/May). In addition, the body-mass index was calculated.

All measurements were taken by the same club’s medical staff, in accordance with guidelines outlined by the ISAK (International Society for the Advancement of Kinanthropometry). Body mass was recorded to the nearest 0.1 kg (Seca 719, Medical Measuring Systems and Scales, Germany) and height was measured with a stadiometer (Seca 213, Medical Measuring Systems and Scales, Germany) to the nearest 0.1 cm. The body-mass index was calculated by dividing body mass by squared height (kilograms per square meter) and used to assess the weight relative to height. Six skinfold thicknesses (triceps, subscapular, supraspinale, abdominal, thigh, and calf) were measured using a skinfold caliper (Harpenden, Holtain Ltd., Crymych, UK). Each measurement was taken three times, in accordance with the recommendations of the Spanish International Group of Kinanthropometry and International Standards for Anthropometric Assessment (ISAK), and the average value for each skinfold calculated. The sum of these six measurements was then calculated (sum of skinfolds). The body fat percentage was estimated using the equation proposed by Yuhasz [27]: body fat (%) = ∑skinfolds · 0.1051 + 2.585.

### 2.3. Statistical Analysis

Descriptive statistics were computed for each anthropometrical measurement (mean ± SD). Practical differences, Cohen’s d effect size [28], were used to compare the anthropometric profile of the players according to period (Period-1 (2008/2009–2012/2013) vs. Period-2 (2013/2014–2017/2018)) and promotion level (non-LFP vs. LFP), as well as separately by playing position. Effect sizes (d) above 0.8, between 0.8 and 0.5, between 0.5 and 0.2, and lower than 0.2 were considered large, moderate, small, and trivial, respectively. We considered effect size values greater than small as worthy of discussion in the results section.

## 3. Results

LFP goalkeepers were largely heavier and taller (effect size = 1.27–1.35) than non-LFP goalkeepers and had moderately greater body fat (effect size = 0.77) than the latter did (Table 1). The goalkeepers in Period-2 were moderately heavier (effect size = 0.52) and largely taller (effect size = 1.13) than the goalkeepers in Period-1 (Table 1).

The body-mass index of the LFP lateral defenders was moderately lower (effect size = 0.53) than that of non-LFP lateral defenders (Table 2). The body-mass index of the LFP central defenders was moderately greater (effect size = 0.54) than that of the non-LFP central defenders (Table 2). The body fat of defenders, lateral defenders, and central defenders in Period-2 was moderately/largely lower (effect size = 0.55–0.95) than that of the same players in Period-1 (Table 2). The lateral defenders in Period-1 were moderately slimmer (effect size = 0.62) and their body-mass index moderately lower (effect size = 0.51) than that of lateral defenders in Period-2 (Table 2).

The weight of the LFP midfielders was moderately greater (effect size = 0.56) than that of non-LFP midfielders; and LFP lateral midfielders were largely heavier and taller (effect size = 1.38–1.98) than non-LFP lateral midfielders (Table 3). The body-mass index of the LFP central midfielders was moderately greater (effect size = 0.51) than that of the non-LFP central midfielders (Table 3). Lateral midfielders in Period-1 were largely taller (effect size = 1.05) than those in Period-2 (Table 3); in turn, central midfielders in Period-2 were moderately slimmer (effect size = 0.63) and mainly shorter (effect size = 0.83) than central midfielders in Period-1 (Table 3).

The LFP attackers were largely heavier (effect size = 0.88) and taller (effect size = 1.20) than the non-LFP attackers (Table 4). The attackers in Period-1 were moderately taller (effect size = 0.66) than the attackers in Period-2 (Table 4).

## 4. Discussion

The aims of this study were to evaluate whether the anthropometric profile of soccer players changed over a decade (i.e., 2008/2009–2017/2018), and to compare the anthropometric profile of the Spanish elite reserve soccer players according to the competitive level they subsequently achieved. We found that changes in the anthropometric profile over time differed by playing position. In the same vein, the relevance of each anthropometric characteristic to promotion to Spanish high-level soccer varied according to playing position. The height of the goalkeepers, lateral midfielders, and attackers increased substantially over time. At the academy level, it appears that being taller is an advantage for goalkeepers, lateral midfielders, and attackers when it comes to being promoted to high-level soccer. Despite the body fat of defenders declining over time, this factor did not determine the competition level attained for any playing position.

We found a substantial decrease (effect size = 0.55–0.95, moderate–large) in body-fat values over time in the defenders, lateral defenders, and central defenders (Table 2). This reduction should be viewed positively, given that body fat levels correlate negatively with aerobic fitness [4] and the physical demands on defenders in matches at high-level have increased substantially [23]. Despite the fact that the physical demands of matches on players have been found to vary substantially between lateral defenders and central defenders [23], it appears that after a continuum selection process in elite soccer academies [29] the body fat of elite reserve defenders is optimal and its decrease is not an advantage for promotion to the Spanish LFP (Table 2). On the other hand, while the body-mass index of the LFP lateral defenders was lower than that of the non-LFP lateral defenders, the body-mass index of the LFP central defenders was greater compared with that of the non-LFP central defenders (Table 2). This suggests that greater weight per height is an advantage for central defenders. Martínez-Santos et al. [12] found that elite Spanish reserve LFP central defenders were moderately smaller than non-LFP central defenders (1.82 ± 0.04 vs. 1.85 ± 0.04; effect size = 0.62) in a similar high-level Spanish reserve team. In contrast, we found trivial differences between both groups (LFP: 1.84 ± 0.03 m vs. non-LPF: 1.84 ± 0.04 m) (Table 2). The moderate differences (1.82 ± 0.04 vs. 1.84 ± 0.03; effect size = 0.50) between the LFP central defenders in both academies suggest that each club assesses the height of their central defenders in different ways.

Taking all midfielders together as a whole, there has been barely any change in anthropometric profile over time. However, when we differentiated between lateral midfielders and central midfielders, several considerable differences could be seen. Furthermore, the evolution of the height of the lateral midfielders and central midfielders over time showed contrasting trends (Table 3). Thus, the generic classification of “midfielders” may be insufficient when it comes to assessing the anthropometric profile of elite reserve players. At the academy level, the increase in height of the lateral midfielders appears to be coherent with the selection of lateral midfielders to Spanish high-level soccer; that is, the LFP lateral midfielders were largely taller (effect size = 1.98) than the non-LFP lateral midfielders (Table 3), suggesting that a greater height is an advantage for lateral midfielders in this regard. In contrast, Martínez-Santos et al. [12] found no substantial differences between LFP and non-LFP lateral midfielders in a very similar soccer academy. Unfortunately, they did not provide the height values so we cannot compare both groups of lateral midfielders [12]. On the other hand, our central midfielders in Period-2 were moderately slimmer (effect size = 0.63) and largely shorter (effect size = 0.83) than the central midfielders in Period-1. However, it seems that being slimmer and shorter than other central midfielders in the elite reserve team is not an advantage when it comes to promotion to Spanish elite soccer, although a higher body-mass index can be. Linking this with the results for the LFP central defenders, we suggest that a greater weight per height can be an advantage for those who play in a central position in the team. In contrast, Martínez-Santos et al. [12] found that the LFP central midfielders in their elite reserve team were moderately taller than their non-LFP central midfielders (effect size = 0.67). The moderate differences (1.82 ± 0.05 vs. 1.78 ± 0.05 m; effect size = 0.67) between the LFP central midfielders in both academies suggest that, as mentioned for central defenders, each club views height in different way (1.82 ± 0.05 vs. 1.78 ± 0.05 m; effect size = 0.67).

The LFP attackers were largely heavier (effect size = 0.88) and taller (effect size = 1.20) than the non-LFP attackers. At the academy level, the moderate increase (effect size = 0.66) in the height of attackers over time suggests a consistent selection process in the reserve team. In their study, Martínez-Santos et al. [12] also found that the LFP attackers (1.81 ± 0.05 vs. 1.77 ± 0.06 m; effect size = moderate) were taller than the non-LFP attackers (although the LFP attackers in our study were moderately taller than the LFP attackers in their high-level reserve team; 1.85 ± 0.05 vs. 1.81 ± 0.05; effect size = moderate). It would appear that here, too, the two soccer academies view the height of those that play in central playing positions (i.e., central defenders, central midfielders, and attackers) in a different way.

A previous study showed that height and body mass in goalkeepers differentiated elite from non-elite junior players (under-19s) (effect size > 0.6) [30]. In addition, Martínez-Santos et al. [12] found that LFP goalkeepers were largely taller (effect size = 1.12) and moderately heavier (effect size = 0.71) than non-LFP goalkeepers [12]. Similarly, we found the LFP goalkeepers to be largely heavier (effect size = 1.35) and taller (effect size = 1.27) than the non-LFP goalkeepers (Table 1). These results suggest that despite being heavier and having more body fat (Table 1), the tallest goalkeepers in high-level reserve teams have an advantage over their teammates (1.90 ± 0.02 vs. 1.86 ± 0.04 m) when it comes to promotion to the Spanish 1st and 2nd divisions. At the academy level, the moderate increase (effect size = 1.13) in the height of goalkeepers over time suggests a consistent selection process in the elite reserve team.

The main limitation of this study was that no other factors were considered, such as the physical fitness performance and socioeconomic status of the players. Despite this, the assessment of the anthropometric profile during a decade in an elite Spanish reserve team could be very interesting for physical coaches and club medical staff.

## 5. Conclusions

The changes in anthropometric profile over time differed by playing position. Similarly, the relevance of each anthropometric characteristic for promotion to Spanish high-level soccer varied according to playing position. The height of goalkeepers, lateral midfielders, and attackers has increased substantially over time. At the academy level, it appears that being taller is an advantage for goalkeepers, lateral midfielders, and attackers in terms of promotion to high-level soccer. Despite the finding that the body fat of defenders has fallen over time, body fat did not determine the competition level attained for any playing position.

The study shows the reference values of the anthropometric profile of elite reserve soccer players in a Spanish first-division club. In addition to other characteristics, it would be interesting to consider the height of players in the selection process of young goalkeepers, lateral midfielders, and attackers. The assessment of the anthropometric profile and the application of interventions by medical staff should be designed according to the playing position of the players.

## Figures and Tables

**Table 1:** Comparison of the anthropometric profile of goalkeepers in Period-1 (2008/2009–2012/2013) with that of goalkeepers in Period-2 (2013/2014–2017/2018) and of Spanish non-LFP players with that of Spanish LFP players (2008/2009–2017/2018).

Variable	Period-1	Period-2	Effect Size	Non-LFP	LFP	Effect Size
	*n* = 5 *	*n* = 7 **		*n* = 9	*n* = 3	
Body mass (kg)	79.38 ± 4.09	81.64 ± 4.59	0.52 (*moderate)*	79.59 ± 4.49	84.03 ± 1.20	1.35 (*large*)
Height (m)	1.85 ± 0.03	1.89 ± 0.04	1.13 (*large*)	1.86 ± 0.04	1.90 ± 0.02	1.27 (*large*)
Body-mass index	23.22 ± 0.50	23.00 ± 1.25	0.21 (*small*)	23.05 ± 1.25	23.22 ± 0.67	0.17 (*trivial*)
Body fat (%)	7.51 ± 1.23	7.22 ± 1.00	0.26 (*small*)	7.16 ± 1.10	7.91 ± 0.83	0.77 (*moderate*)

Non-LFP: players who had never competed in the Spanish first or second division; LFP: players who had signed a contract with a Spanish first- or second-division team and who had played at least one match as a full-time professional. * Non-LFP players: 3, LFP players: 2; ** Non-LFP players: 6, LFP players: 1.

**Table 2:** Comparison of the anthropometric profile of defenders in Period-1 (2008/2009–2012/2013) with that of defenders in Period-2 (2013/2014–2017/2018) and of Spanish non-LFP players with that of Spanish LFP players (2008/2009–2017/2018).

Variable	Period-1	Period-2	Effect Size	Non-LFP	LFP	Effect Size
*Defenders*	*n* = 16 *	*n* = 19 **		*n* = 20	*n* = 15	
Body mass (kg)	75.59 ± 5.26	73.76 ± 5.29	0.35 (*small)*	74.87 ± 5.48	74.24 ± 5.17	0.12 (*trivial*)
Height (m)	1.79 ± 0.07	1.78 ± 0.06	0.15 (*trivial*)	1.78 ± 0.07	1.79 ± 0.05	0.16 (*trivial*)
Body-mass index	23.69 ± 1.46	23.17 ± 0.83	0.44 (*small*)	23.53 ± 1.32	23.24 ± 0.96	0.25 (*small*)
Body fat (%)	7.42 ± 0.95	6.79 ± 0.76	0.73(*moderate*)	6.99 ± 0.86	7.19 ± 0.97	0.22 (*small*)
*Lateral defenders*	*n =* 9 ^	*n* = 12 ^^		*n* = 11	*n* = 10	
Body-mass (kg)	73.24 ± 4.39	70.85 ± 3.31	0.62 (*moderate)*	72.10 ± 4.05	71.63 ± 3.92	0.12 (*trivial*)
Height (m)	1.75 ± 0.06	1.75 ± 0.03	0.00 (*trivial*)	1.74 ± 0.04	1.76 ± 0.04	0.50 (*small*)
Body-mass index	23.97 ± 1.90	23.22 ± 0.89	0.51 (*moderate*)	23.89 ± 1.61	23.15 ± 1.14	0.53 (*moderate*)
Body fat (%)	7.24 ± 0.88	6.76 ± 0.88	0.55 (*moderate*)	6.83 ± 0.88	7.11 ± 0.94	0.31 (*small*)
*Central defenders*	*n* = 7 *^#^*	*n* = 7 ^##^		*n* = 9	*n* = 5	
Body-mass (kg)	78.62 ± 4.95	78.74 ± 4.24	0.03 (*trivial)*	78.24 ± 5.23	79.46 ± 2.79	0.30 (*small*)
Height (m)	1.84 ± 0.04	1.85 ± 0.03	0.28 (*small*)	1.84 ± 0.04	1.84 ± 0.03	0.00 (*trivial*)
Body-mass index	23.32 ± 0.51	23.09 ± 0.78	0.35 (*small*)	23.09 ± 0.71	23.42 ± 0.50	0.54 (*moderate*)
Body fat (%)	7.65 ± 1.06	6.84 ± 0.57	0.95 (*large*)	7.19 ± 0.84	7.34 ± 1.13	0.15 (*trivial*)

Non-LFP: players who had never competed in the Spanish first or second division; LFP: players who had signed a contract with a Spanish first- or second-division team and who had played at least one match as a full-time professional. * Non-LFP players: 10, LFP players: 6; ** Non-LFP players: 10, LFP players: 9; ^ Non-LFP players: 5, LFP players: 4; ^^ Non-LFP players: 6 LFP players: 6; *^#^* Non-LFP players: 5, LFP players: 2; *^##^* Non-LFP players, 4; LFP players: 3.

**Table 3:** Comparison of the anthropometric profile of midfielders in Period-1 (2008/2009–2012/2013) with that of midfielders in Period-2 (2013/2014–2017/2018) and of Spanish non-LFP players with that of Spanish LFP players (2008/2009–2017/2018).

Variable	Period-1	Period-2	Effect Size	Non-LFP	LFP	Effect Size
*Midfielders*	*n* = 13 *	*n* = 23 **		*n* = 20	*n* = 16	
Body mass (kg)	72.65 ± 6.88	70.58 ± 6.01	0.32 (*small)*	69.82 ± 6.71	73.21 ± 5.42	0.56 (*moderate*)
Height (m)	1.78 ± 0.07	1.77 ± 0.06	0.15 (*trivial*)	1.76 ± 0.07	1.78 ± 0.05	0.33 (*small*)
Body-mass index	22.88 ± 1.21	22.65 ± 1.73	0.15 (*trivial*)	22.46 ± 0.92	23.08 ± 2.08	0.39 (*small*)
Body fat (%)	6.98 ± 0.50	7.04 ± 0.80	0.09 (*trivial*)	7.01 ± 0.75	7.02 ± 0.64	0.01 (*trivial*)
*Lateral midfielders*	*n* = 5 ^	*n* = 10 ^^		*n* = 10	*n* = 5	
Body mass (kg)	67.42 ± 3.43	69.00 ± 5.22	0.36 (*small*)	66.73 ± 4.32	71.97 ± 3.19	1.38 (*large*)
Height (m)	1.72 ± 0.02	1.76 ± 0.05	1.05 (*large*)	1.72 ± 0.03	1.79 ± 0.04	1.98 (*large*)
Body-mass index	22.78 ± 0.75	22.34 ± 1.13	0.46 (*small*)	22.48 ± 1.03	22.49 ± 1.09	0.01 (*trivial*)
Body fat (%)	6.96 ± 0.56	7.02 ± 0.86	0.08 (*trivial*)	6.97 ± 0.81	7.07 ± 0.70	0.13 (*trivial*)
*Central midfielders*	*n* = 8 ^#^	*n* = 13 ^##^		*n* = 10	*n* = 11	
Body mass (kg)	75.91 ± 6.54	71.80 ± 6.49	0.63 (*moderate*)	72.92 ± 7.42	73.77 ± 6.24	0.12 (*trivial*)
Height (m)	1.82 ± 0.06	1.77 ± 0.06	0.83 (*large*)	1.80 ± 0.08	1.78 ± 0.05	0.30 (*small*)
Body-mass index	22.95 ± 1.47	22.90 ± 2.10	0.03 (*trivial*)	22.44 ± 0.84	23.35 ± 2.39	0.51 (*moderate*)
Body fat (%)	7.00 ± 0.49	7.05 ± 0.79	0.08 (*trivial*)	7.06 ± 0.74	7.01 ± 0.65	0.07 (*trivial*)

Non-LFP: players who had never competed in the Spanish first or second division; LFP: players who had signed a contract with a Spanish first- or second-division team and who had played at least one match as a full-time professional.* Non-LFP players: 7, LFP players: 6; ** Non-LFP players: 13, LFP players: 10; *^* Non-LFP players: 3, LFP players: 2; *^^* Non-LFP players: 7, LFP players: 3; *^#^* Non-LFP players: 4, LFP players: 4; *^##^* Non-LFP players: 6; LFP players: 7.

**Table 4:** Comparison of the anthropometric profile of attackers in Period-1 (2008/2009–2012/2013) with that of attackers in Period-2 (2013/2014–2017/2018) and of Spanish non-LFP players with that of Spanish LFP players (2008/2009–2017/2018).

Variable	Period-1	Period-2	Effect Size	Non-LFP	LFP	Effect Size
	*n* = 8 *	*n* = 7 **		*n* = 8	*n* = 7	
Body mass (kg)	75.32 ± 5.46	77.23 ± 6.62	0.32 (*small*)	73.93 ± 4.57	78.82 ± 6.44	0.88 (*large*)
Height (m)	1.80 ± 0.05	1.84 ± 0.07	0.66 (*moderate*)	1.79 ± 0.05	1.85 ± 0.05	1.20 (*large*)
Body-mass index	23.16 ± 1.08	22.90 ± 1.21	0.23 (*small*)	23.10 ± 1.24	22.97 ± 1.04	0.11 (*trivial*)
Body fat (%)	7.31 ± 1.47	7.34 ± 0.97	0.02 (*trivial*)	7.41 ± 1.20	7.23 ± 1.32	0.14 (*trivial*)

Non-LFP: players who had never competed in the Spanish first or second division; LFP: players who had signed a contract with a Spanish first- or second-division team and who had played at least one match as a full-time professional. * Non-LFP players: 4, LFP players 4; ** Non-LFP players: 4, LFP players: 3.
